# Multimodal PET/MR imaging of prolonged disorders of consciousness: a pilot feasibility study

**DOI:** 10.3389/fnins.2026.1761097

**Published:** 2026-02-20

**Authors:** Ning Sun, YiWei Liu, Hua Lin, Jing Xiong, Yi He, HuiLin Yang, MingGang Su, Jing He

**Affiliations:** 1 Rehabilitation Medicine Center and Institute of Rehabilitation Medicine, West China Hospital, Sichuan University, Chengdu, China; 2 Key Laboratory of Rehabilitation Medicine in Sichuan Province, West China Hospital, Sichuan University, Chengdu, China; 3 Department of Nuclear Medicine, West China Hospital, Sichuan University, Chengdu, China; 4 Department of Rehabilitation Medicine, West China Tianfu Hospital, Sichuan University, Chengdu, China; 5 Acupuncture and Tuina School, Chengdu University of Traditional Chinese Medicine, Chengdu, China

**Keywords:** functional connectivity, PET/MR imaging, posterior cingulate cortex, prolonged disorders of consciousness, visual network

## Abstract

**Background:**

Prolonged disorders of consciousness (pDOC), including vegetative/unresponsive wakefulness state (VS/UWS) and minimally conscious state (MCS), pose significant diagnostic and prognostic challenges. Multimodal neuroimaging has emerged as a promising tool to uncover neural biomarkers that reflect residual brain function and guide management. This pilot feasibility study aimed to preliminarily characterize metabolic, functional, and structural brain alterations in pDOC patients using simultaneous positron emission tomography/magnetic resonance (PET/MR) imaging, and to examine their tentative associations with clinical behavioral responsiveness.

**Methods:**

Eight pDOC patients and eight matched healthy controls underwent hybrid 18F-FDG PET/MR scanning. Resting-state fMRI, diffusion tensor imaging (DTI), and FDG-PET were processed to assess amplitude of low-frequency fluctuations (ALFF), fractional anisotropy (FA), and glucose metabolism, respectively. Group differences were analyzed, and correlations with Coma Recovery Scale-Revised (CRS-R) scores were evaluated. Multimodal integration was performed across imaging modalities.

**Results:**

Compared to controls, pDOC patients exhibited reduced ALFF and FDG uptake in the posterior cingulate cortex (PCC) and anterior cingulate cortex (ACC), with exploratory increased ALFF in the visual cortex inversely correlated with visual responsiveness. Functional connectivity analyses revealed attenuated intra- and inter-network connectivity in the DMN, SN, and DAN. FDG-PET identified metabolic hypofunction in the insula, frontal cortex, and cerebellum, while DTI demonstrated widespread white matter FA reductions. Multimodal correspondence revealed partially overlapping abnormalities in the PCC and occipital regions, highlighting candidate hubs that may be relevant to consciousness level and warrant further validation.

**Conclusion:**

Simultaneous FDG-PET/MR was feasible in this pilot pDOC cohort and provided a convergent, multimodal assessment of metabolic-functional-structural alterations. The PCC and occipital visual cortices emerge as key regions linked to consciousness levels. Given the small sample size and cross-sectional design, these findings are preliminary, and warrant validation in larger longitudinal cohorts before clinical translation.

## Introduction

Prolonged disorders of consciousness (pDOC) are defined as disorders of consciousness persisting for more than 28 days following severe brain injury (Kondziella et al., 2020). With the advancement of emergency medical techniques improving survival rates after severe brain injury, the incidence of pDOC has shown a consistent annual increase (Wan et al., 2024). These conditions primarily manifest in severe cases of central nervous system damage, such as stroke, trauma, and hypoxic encephalopathy (Kondziella et al., 2020; Wan et al., 2024). The clinical and socioeconomic burden of pDOC is substantial, as patients frequently develop multiple complications, including hydrocephalus, pulmonary infections, urinary tract infections, and paroxysmal sympathetic hyperactivity (Wan et al., 2024). In the United States, epidemiological studies reported approximately 35,000 patients in persistent vegetative state / unresponsive wakefulness syndrome (VS/UWS) (Multi-Society Task Force on Pvs, 1994) and 390,000 patients in minimally conscious state (MCS) (Strauss et al., 2000). In Europe and Japan, the prevalence ranged from 0.2 to 3.4 per 100,000 inhabitants for VS and 1.5 per 100,000 for MCS (Pisa et al., 2014). According to nationwide statistics in China, the number of pDOC patients has exceeded 500,000, with an annual incidence of 70,000–100,000 cases and medical costs of 30–50 billion yuan (He, 2018). The significant burden and complexity of pDOC necessitate better understanding of its underlying neural mechanisms (Thibaut et al., 2019).

Recent studies have emphasized that objective biomarkers identified through advanced neuroimaging techniques are crucial for assessing consciousness levels and predicting potential outcomes (Estraneo et al., 2020). Alterations in brain metabolism and network function serve as critical biological markers for pDOC, directly linking to patients’ clinical manifestations and recovery potential. Studies using multimodal neuroimaging have revealed specific disruption patterns in pDOC patients compared to healthy individuals. For instance, 18-Fluorodeoxyglucose Positron Emission Tomography (FDG-PET) studies have demonstrated significant metabolic changes in specific brain regions, particularly showing reduced metabolism in the right parahippocampal cortex, right precuneus, and bilateral middle cingulate cortex (Stender et al., 2015). These metabolic patterns show significant correlations with clinical consciousness scores, with lower metabolism in these regions associated with poorer outcomes on the Coma Recovery Scale-Revised (CRS-R) (Cavaliere et al., 2018). In terms of brain functional organization, resting-state functional MRI studies have identified disruptions in critical neural networks. The default mode network (DMN), which includes regions like the medial prefrontal cortex and posterior cingulate/precuneus, shows distinct connectivity patterns based on consciousness levels (Demertzi et al., 2014; Vanhaudenhuyse et al., 2010). In UWS patients, the medial prefrontal region shows no activation during expected arousal periods, while MCS patients exhibit reduced but present activity compared to healthy control (HC) (Crone et al., 2011). These findings suggest that both metabolic activity and network connectivity could serve as potential biomarkers for differentiating between consciousness states (Stender et al., 2014).

The integration of metabolic and functional connectivity data through hybrid Positron Emission Tomography/Magnetic Resonance (PET/MR) imaging presents a powerful approach for investigating differences between pDOC patients and healthy individuals. This simultaneous acquisition reduces potential confounding factors, as temporal alignment of metabolic and functional information is maintained (Shiyam Sundar et al., 2020). Studies have shown that multiple critical networks, including the DMN, Salience Network (SN), Dorsal Attention Network (DAN), and Auditory Network (AN), can differentiate between consciousness states with high accuracy (Chennu et al., 2017; Porcaro et al., 2021). Particularly, DMN connectivity patterns demonstrate strong diagnostic value (Coppola et al., 2022).

Through simultaneous PET/MR imaging, this pilot study aimed to provide preliminary insights into the neural mechanisms of prolonged disorders of consciousness by addressing three exploratory objectives:

(i) To characterize differential metabolic patterns between pDOC patients and healthy controls in specific brain regions;

(ii) To describe distinctive features of functional network connectivity in pDOC;

(iii) To explore associations between these imaging measures and clinical manifestations, with particular focus on residual responsiveness.

The study was not powered to establish validated biomarkers or to test clinical efficacy. Despite these constraints, the work illustrates the practical feasibility of simultaneous PET/MR in severely brain-injured patients and provides an integrated description of metabolic, functional and structural abnormalities in pDOC. These preliminary patterns can help to prioritize candidate regions and modalities for future longitudinal and multicentre studies.

## Materials and methods

### Study population

This was a single-center pilot feasibility study using simultaneous PET/MR in patients with pDOC and HCs. The study was approved by the Biomedical Research Ethics Committee of West China Hospital of Sichuan University (approval no. 20201070) and performed according to the ethical principles introduced in 1964 by the Declaration of Helsinki and its later amendments.

We screened for the study pDOC patients consecutively admitted to the Department of Rehabilitation Medicine at West China Hospital, in Chengdu (China) from January 2021 to September 2022, fulfilling the following inclusion criteria: (1) clinical diagnosis of coma, VS/UWS, or MCS according to standard diagnostic criteria; (2) onset > 28 days; (3) right-handed; (4) age 18–75 years; (5) stable vitals, not in other trials; (6) informed consent from legal representative. We excluded from the study patients with: (1) intracranial infections or brain death; (2) psychiatric disorders, drug abuse, or alcohol addiction history; (3) prior neurological diseases (e.g., neurodegeneration, stroke); (4) unstable respiratory/circulatory conditions; (5) severe endocrine/metabolic diseases or significant comorbidities; (6) status epilepticus; (7) pregnancy, multiple traumas, or major skin defects; (8) contraindications to PET/MR. For each patient, etiology and the interval between injury onset and PET/MR acquisition (days post-injury) were recorded.

In addition, 8 normal subjects with brain PET/MR matched for gender, age, and right-handedness were recruited as HC. The HC had no cognitive impairment complaint; no history of any neurological or psychiatric disorders, chronic diseases, or tumors; and no relevant family history.

### PET/MR acquisition protocol

Brain neuroimaging was conducted using a 3.0T integrated PET/MR scanner (GE Healthcare). Dynamic PET imaging began after an intravenous bolus injection of 18F-fluorodeoxyglucose (18F-FDG) at a dose of 5 MBq/Kg. Subjects fasted for at least 6 h before the scan, and blood glucose levels were checked upon arrival at the PET center. FDG was administered only if blood glucose levels were under 120 mg/dL. Pillows and fixation foam were used to minimize head movements within the scanner, and earplugs and eye masks were provided to reduce noise and light interference. PET/MR scans were conducted in the morning following routine nursing procedures. CRS-R assessments were performed by two trained, independent raters using duplicate evaluations, completed within 12 h prior to PET/MR acquisition; discrepant ratings were resolved by consensus. And vital signs were continuously monitored via a bedside monitor visible through the glass during the scan. If necessary, a family member accompanied the patient. The scan was immediately stopped if the patient showed agitation or significant abnormalities in vital signs. Peri-scan zolpidem administration (timing/dose) was recorded and is summarized at the individual level in Supplementary Table S1.

The PET/MR scanning protocol involved the following steps: (1) Localization Scans: Localization images were initially obtained to determine the appropriate scanning field of view for the head. Scans were conducted in sagittal, coronal, and horizontal planes to ensure proper alignment. (2) T1-Weighted Structural Imaging: High-resolution 3D T1-weighted images were acquired with the following parameters: repetition time (TR) = 2,700 ms, echo time (TE) = 3.39 ms, field of view (FOV) = 256 × 256 mm, matrix size = 256 × 256, flip angle (FA) = 7°, planar resolution = 1 mm, and slice thickness = 1 mm. (3) Resting-State Functional MRI (BOLD-fMRI): Resting-state scans used a GRE-EPI sequence performed simultaneously with PET, with parameters: TR = 2,000 ms, TE = 30 ms, FOV = 240 × 240 mm, matrix = 64 × 64, FA = 90°, slices = 30, and slice thickness = 5 mm. (4) Diffusion Tensor Imaging (DTI): Acquired using an EPI sequence with 30 non-collinear diffusion directions (*b* = 1,000 s/mm^2^) and one non-weighted (*b* = 0) image. Parameters: TR = 17,000 ms, TE = 98 ms, FA = 90°, FOV = 256 × 256 mm, matrix = 128 × 128, slices = 78, and slice thickness = 2 mm. Three acquisitions were averaged for improved signal-to-noise ratio. (5) PET Imaging: Conducted simultaneously with fMRI, with parameters: slice thickness = 3 mm, slice interval = 1.5 mm, and matrix = 256 × 256.

### Imaging processing and analysis

#### Data processing

Resting-state fMRI data were preprocessed using SPM12 (Wellcome Department of Imaging Neuroscience, London, United Kingdom)^1^ running in MATLAB R2018a (MathWorks, Natick, MA, United States). For each subject, the first 10 volumes were discarded to allow for signal stabilization. The remaining images underwent slice-timing correction and rigid-body realignment. The mean functional image was co-registered to the individual T1-weighted structural image, which was then normalized to the Montreal Neurological Institute (MNI) space. The normalization parameters were applied to the functional images and resampled to 3 × 3 × 3 mm^3^ voxel size. Images were spatially smoothed with a 6-mm full-width at half-maximum Gaussian kernel. A linear trend was removed, and nuisance covariates (24 head motion parameters, mean white-matter signal and mean cerebrospinal fluid signal) were regressed out. Finally, temporal band-pass filtering (0.01–0.08 Hz) was applied. After preprocessing, amplitude of low-frequency fluctuation (ALFF) maps were computed in the standard way and normalized by the global mean ALFF within a brain mask.

Head motion was quantified using framewise displacement (FD) derived from the realignment parameters. Subjects with mean FD > 0.5 mm or maximum displacement > 3 mm in any direction were predefined as exclusion criteria. No subject met these criteria. All preprocessed images were visually inspected to confirm the absence of major artifacts or misregistration.

DTI data were processed using the FMRIB Software Library (FSL).^2^ First, nonbrain tissue was removed using the Brain Extraction Tool (BET). Eddy-current-induced distortions and simple head motion were corrected using eddy. A diffusion tensor model was then fitted at each voxel with DTIFIT to generate fractional anisotropy (FA) maps. Voxelwise statistical analysis of FA was performed using tract-based spatial statistics (TBSS). All FA images were nonlinearly registered to the FMRIB58_FA template, averaged, and skeletonised. A mean FA skeleton was created and thresholded at FA > 0.2 to include major white-matter tracts. Individual FA data were projected onto this skeleton for subsequent group comparisons.

PET data were preprocessed using SPM12. All PET images underwent attenuation, scatter and decay correction on the scanner. Dynamic frames were realigned and summed to obtain a static FDG image representing uptake during the resting-state acquisition. Each PET volume was co-registered to the individual T1-weighted MRI, spatially normalized to MNI space using the same deformation field as the structural image, and smoothed with a 6-mm FWHM Gaussian kernel. To account for inter-individual variability in global tracer uptake, voxelwise values were intensity-normalized by the global mean uptake within the brain to produce standardized uptake value ratio (SUVR) maps. Global normalization was chosen rather than a regional reference because of the heterogeneous lesion distribution and to remain consistent with previous FDG-PET studies in pDOC.

#### Statistical and network analysis

Group-level statistical analyses were performed in SPM12 and FSL. For voxel-wise ALFF and FDG-PET contrasts between pDOC patients and healthy controls, we used two-sample *t*-tests with a cluster-forming threshold of *p* < 0.001 (uncorrected) and cluster-level family-wise error (FWE) correction at *p* < 0.05. For DTI, voxel-wise analysis of FA maps was carried out using tract-based spatial statistics (TBSS) with threshold-free cluster enhancement (TFCE) and family wise error control at pTFCE < 0.05. At the network level, intra- and inter-network functional connectivity values were compared between groups using two-sample *t*-tests. Multiple comparisons across network pairs were controlled using the false discovery rate (FDR) at *q* < 0.05. To account for the small sample size, non-parametric permutation tests (5,000 permutations) were additionally conducted for the main between-group contrasts in ALFF, FDG-PET, and FA.

The association between clinical measures (CRS-R total and subscale scores) and neuroimaging parameters (ALFF values, FDG uptake and FA values) was evaluated using Pearson’s correlation coefficients. Correlations were restricted to regions and tracts that showed significant between-group differences. Because this introduces a degree of circularity, these correlations were treated as exploratory, were not corrected for multiple comparisons, and were not used to support definitive inferential claims.

#### Multimodal integration

Spatial correspondence between functional, metabolic, and structural alterations was evaluated using standardized MNI coordinates. Regions showing significant changes across multiple modalities were identified and categorized according to their network affiliations. To validate functional and metabolic findings, we performed cross-modal correlation analyses between ALFF values and FDG uptake in regions showing significant group differences. The relationship between structural connectivity (FA values) and functional connectivity was assessed for major white matter tracts and their associated functional networks. This multimodal approach enabled comprehensive characterization of brain alterations in pDOC patients across different neurobiological dimensions. Multiple comparison corrections were applied using family-wise error (FWE) correction for fMRI and PET analyses, and TFCE correction for DTI analyses. Effect sizes were calculated using Cohen’s *d* for between-group comparisons. For all analyses, the significance level was set at *p* < 0.05 after correction for multiple comparisons. Results were visualized using BrainNet Viewer and FSLview for structural and functional findings, respectively.

## Results

### Demographic and clinical characteristics

PET/MR were obtained in 8 pDOC patients (6 males, 2 females) with a mean age of 50.1 years (SD = 8.5), see Table 1 for demographic and clinical data. And we recruited eight healthy volunteers (5 males, 3 females) as the control group, with a mean age of 52.0 years (SD = 7.3). The pDOC cohort was etiologically heterogeneous, and days post-injury at the time of PET/MR acquisition varied across patients.

**TABLE 1:** Clinical data of the eight patients.

Clinical features	COMA1	COMA2	COMA3	COMA4	UWS	MCS1	MCS2	MCS3
Age/gender	52	57	48	45	59	33	50	57
Gender	Female	Male	Male	Male	Male	Male	Male	Female
Cause	ICH	ICH	Brain tumor	ICH	HIE	ICH	ICH	Brain tumor
Time of PET/MR (days post-injury)	29	45	36	68	29	90	97	129
CRS-R	4	3	4	6	7	12	10	13

ICH, Intracerebral Hemorrhage; HIE, Hypoxic-Ischemic Encephalopathy; CRS-R, Coma Recovery Scale-Revised.

### Local brain activity and functional network connectivity

**FIGURE 1:**
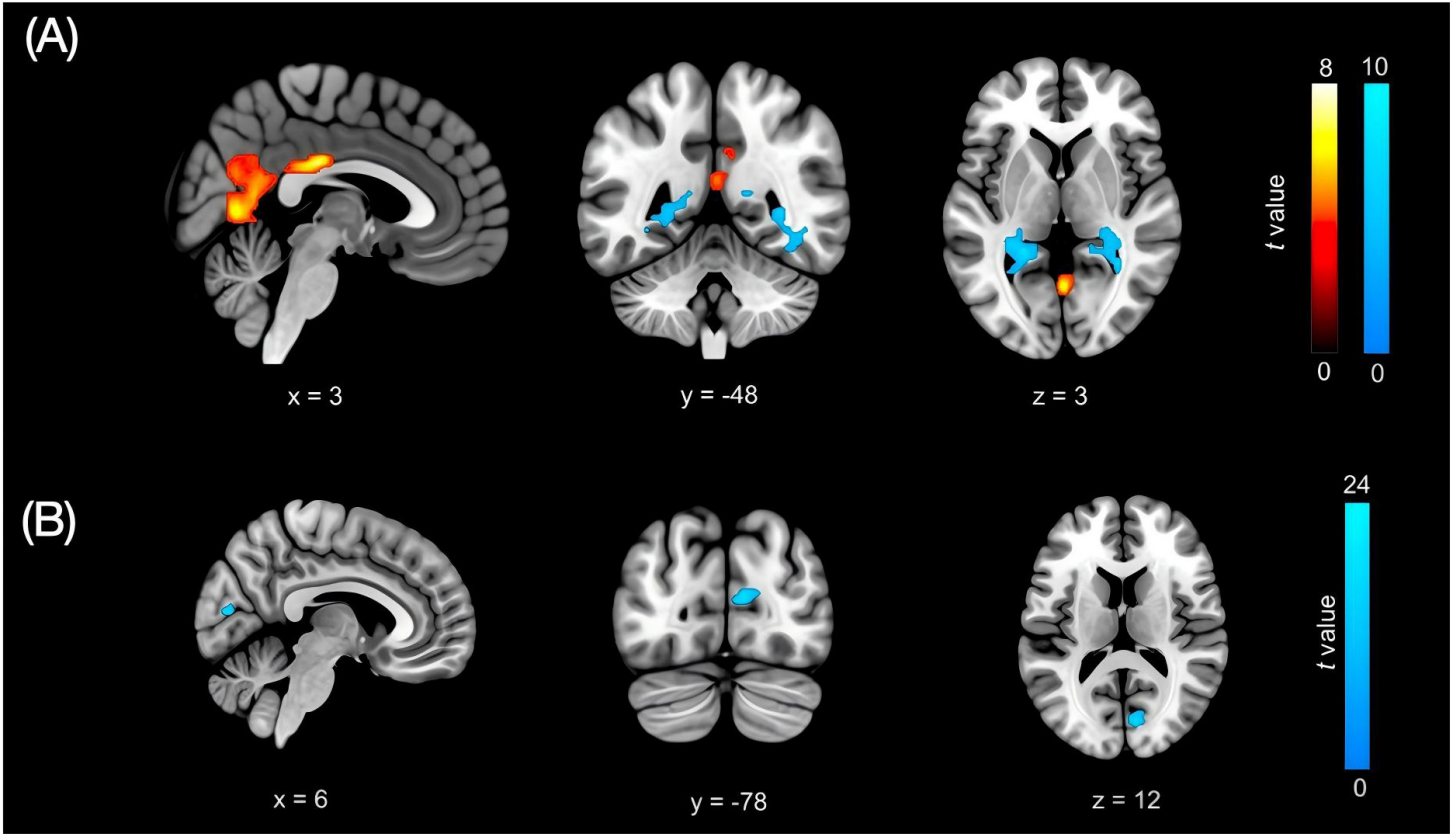
Group differences in ALFF. **(A)** Group differences in ALFF. Warm colors indicate higher ALFF in HC; cool colors indicate higher ALFF in pDOC patients. **(B)** Negative correlation between ALFF and CRS-R visual subscale in pDOC patients (blue regions).

Whole-brain ALFF analysis revealed marked differences between HC and patients with pDOC (Figure 1A). The HC group showed significantly higher ALFF in the right posterior cingulate cortex (PCC; DMN; MNI 3, –24, 27; *t* = 7.84), the midline lingual gyrus (VIS; MNI 0, –63, 6; *t* = 7.23), and the right precuneus (DMN; MNI 3, –54, 12; *t* = 5.61). Conversely, the pDOC group exhibited higher ALFF in the right calcarine cortex (VIS; MNI 33, –48, 3; *t* = 9.86) and the left hippocampus (LN; MNI –21, –42, 3; *t* = 9.46). Within the pDOC patients, ALFF in the left calcarine cortex (VIS; MNI 6, –78, 12; *t* = 23.53) (Figure 1B) was negatively correlated with CRS-R visual subscale scores, suggesting that heightened intrinsic activity in primary visual cortex is associated with poorer overt visual responsiveness in this patient population. However, this correlation is exploratory and does not necessarily indicate preserved visual function.

**FIGURE 2:**
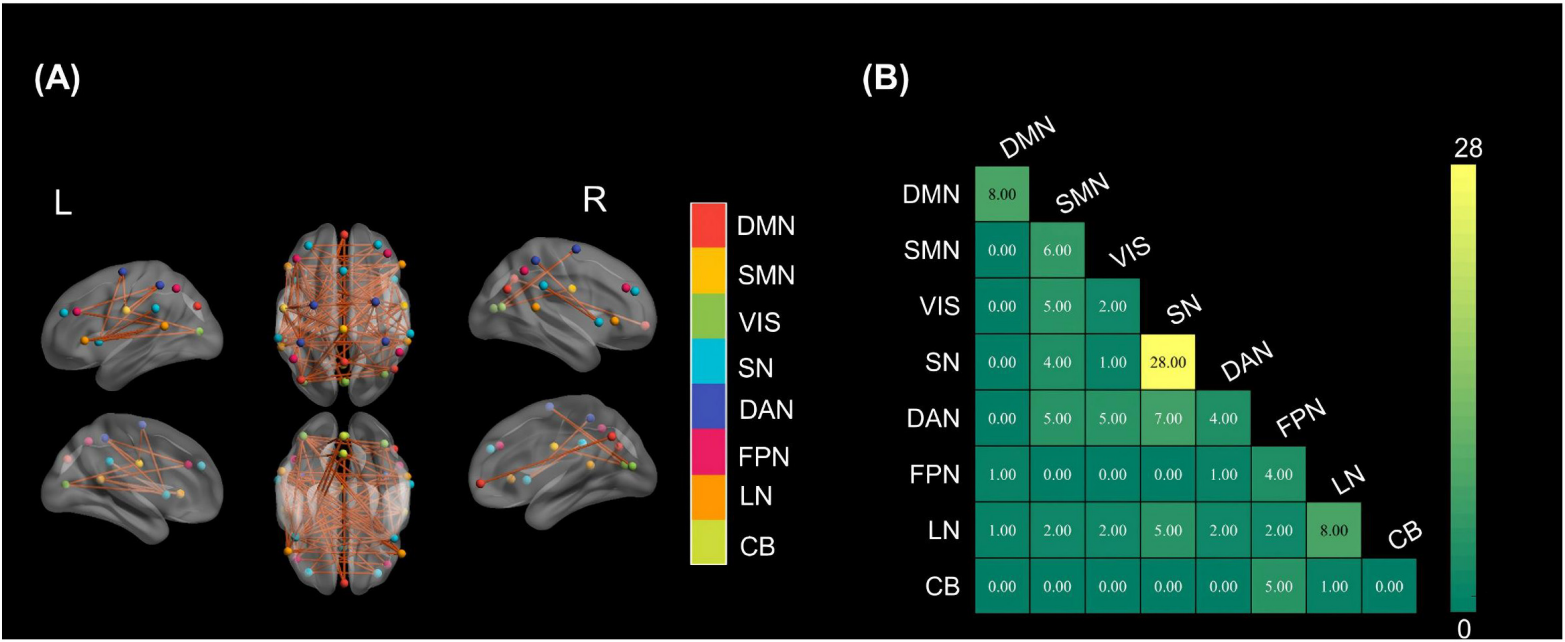
Group differences in FC. **(A)** Brain regions with significantly stronger connections in HC than patients. Node colors denote resting-state networks; orange lines indicate enhanced interregional links. **(B)** Network-wise matrix of stronger connections (control > patient).

There are significant differences in FC between HC and patients. As shown in Figure 2A, healthy individuals exhibited a greater number of stronger FCs, spatially distributed across multiple cortical regions. These enhanced connections involved key hubs of major resting-state networks, including the DMN, DAN, and SN. The corresponding network-wise summary in Figure 2B demonstrated that the SN exhibited the highest degree of intra-network connectivity advantage (28 connections) in controls compared to patients. Additionally, the DAN, DMN, and SMN showed moderate levels of increased connectivity, both within and across networks. In contrast, the VIS and CB demonstrated minimal differences, indicating relatively preserved in pDOC patients.

### Brain metabolic characteristics and white matter integrity

**FIGURE 3:**
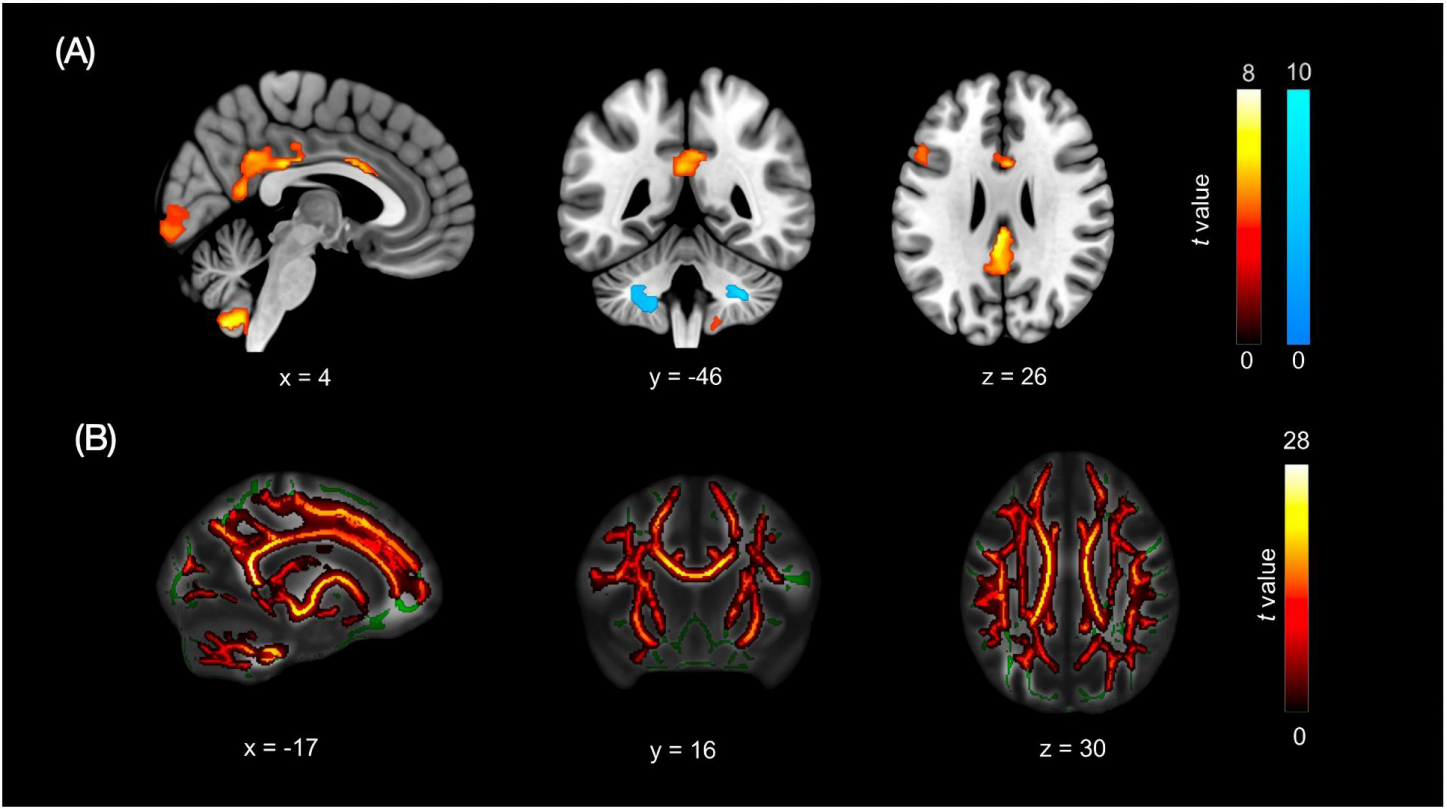
Metabolic and white-matter alterations in pDOC compared with HC. **(A)** FDG-PET glucose metabolism. Warm clusters correspond to higher uptake in controls, whereas cool clusters correspond to higher uptake in pDOC. **(B)** TBSS findings. Warm clusters identify regions with lower FA in pDOC.

FDG-PET revealed a distinct metabolic imbalance between HC and pDOC patients (Figure 3A). Compared to the pDOC, the HC exhibited higher metabolic activity in the following regions: the left insula (SN; MNI −38, 20, −6; *t* = 8.36), the PCC (FPN; MNI 0, −24, 28; *t* = 8.13), the right cerebellum (MNI 32, −76, −52; *t* = 7.98; and 4, −60, −56; *t* = 7.77), the left inferior frontal gyrus (FPN; *t* = 6.68), and the right anterior cingulate cortex (ACC; *t* = 6.24). Conversely, the pDOC showed higher metabolic activity in specific regions of the bilateral cerebellum (right: MNI 30, −46, −42; *t* = 10.36; left: MNI −32, −56, −44; *t* = 8.35).

Diffusion-based TBSS identified one extensive suprathreshold cluster (69,688 voxels, pTFCE < 0.05) of reduced FA in pDOC (Figure 3B). This cluster spanned the bilateral anterior thalamic radiations (MNI –17, 16, 30; *t* = 30.3), corticospinal tracts, cingulum bundles, forceps major, bilateral inferior and superior longitudinal fasciculi, inferior fronto-occipital fasciculi, and uncinate fasciculi, with no white-matter tract showing higher FA in pDOC than in HC.

**FIGURE 4:**
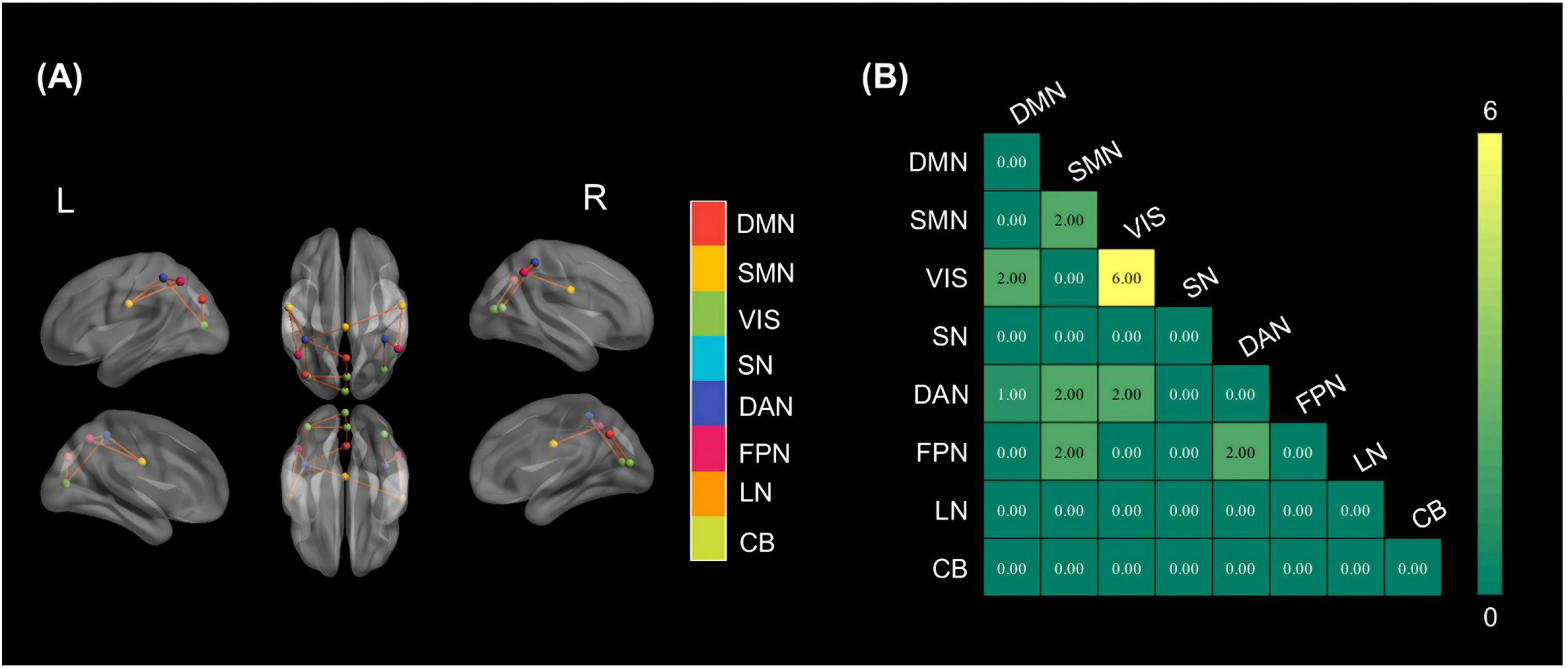
Group differences in structural connectivity. **(A)** Brain regions with significantly stronger structural connections in HC than in patients. Node colors denote resting-state networks; orange lines indicate enhanced interregional tracts. **(B)** Network-wise matrix of stronger structural connections (control > patient).

Structural connectome results are summarized in Figures 4A,B. Within-network alterations were most prominent in the VIS, which contained six significantly different intra-network edges (Figure 4B). Inter-network changes were modest. Two altered edges were detected for a few pairs, chiefly those linking the SMN with VIS, the FPN and the DAN, as well as between VIS and DAN.

Across modalities, we observed partial spatial correspondence between functional and metabolic alterations. Notably, the PCC and occipital/visual regions showed overlapping abnormalities across FDG-PET and resting-state fMRI, highlighting convergent candidate hubs potentially relevant to consciousness level. Individual PET maps co-registered to structural MRI are provided in Supplementary Figure S1 to facilitate case-level visualization.

## Discussion

This pilot feasibility study suggests that simultaneous PET/MR can detect anatomically specific network abnormalities in pDOC. This study shows that simultaneous PET/MR detects consistent but anatomically specific network abnormalities in prolonged disorders of consciousness. The visual system exhibited concordant metabolic, functional, and structural deficits, underlining its role in residual visual responsiveness. The posterior cingulate cortex—at the crossroads of the default mode and salience networks—displayed parallel reductions in FDG uptake and spontaneous BOLD activity, reinforcing its importance for awareness. Region-dependent metabolic increases and decreases in the cerebellum may signal compensatory reorganization. TBSS revealed widespread loss of FA, yet only a subset of these tracts showed corresponding functional disconnection, indicating partial structure-function dissociation.

### Functional-metabolic changes in cingulate cortices

Both the PCC and ACC exhibit parallel reductions in intrinsic activity and glucose metabolism in patients with pDOC. These midline structures are key hubs of the DMN and SN, and are consistently hypoactive in unconscious states. FDG-PET studies have revealed markedly decreased glucose uptake in the FPN (including the midline PCC/precuneus and medial frontal regions) in UWS, whereas patients in a MCS show relatively preserved metabolism in these areas (Dagnino et al., 2024). Similarly, resting-state fMRI indices such as ALFF and fALFF are significantly reduced in pDOC. For instance, fALFF within the dorsal DMN (anchored by the PCC) and anterior SN (encompassing the ACC) is most diminished in UWS compared to MCS (Wang et al., 2022). However, in rare instances, abnormal hyperactivation has been reported in key arousal-related regions in traumatic brain injury (TBI) -related UWS. Notably, Qin et al. (2024) found increased ALFF in the ACC and thalamus prior to spinal cord stimulation, a finding interpreted as a possible compensatory attempt to re-engage arousal circuits. The posterior and anterior cingulate cortices generally exhibit concurrent declines in metabolic and functional measures in pDOC, underscoring their pivotal role in maintaining consciousness and the profound disruption of the DMN and SN in these patients (Crone et al., 2015).

### Visual cortex activity and CRS-R visual function

In this pilot study, ALFF was abnormally elevated in the left calcarine cortex and showed a significant negative correlation with CRS-R visual subscores. Higher intrinsic activity in primary visual cortex was thus associated with poorer overt visual responsiveness. Structural connectivity between the VIS and the SN also showed marked between-group differences. These findings suggest that disturbed integration between sensory and higher-order regulatory systems may be relevant for consciousness in pDOC and are broadly consistent with prior resting-state fMRI work highlighting the role of lower-order networks, including occipital visual cortices, in pDOC (Li et al., 2025; Medina et al., 2022; Wu et al., 2020). Importantly, increased ALFF should not be interpreted as preserved visual processing. Resting-state BOLD amplitude can be influenced by non-neural factors (e.g., physiological noise, residual motion effects, and arousal fluctuations), and may reflect disorganized intrinsic activity in a partially deafferented visual system.

A plausible mechanistic explanation is a disturbance of local excitatory-inhibitory balance in a de-afferented sensory cortex. Experimental data indicate that chronic loss of thalamocortical or sensory input can induce homeostatic up-regulation of spontaneous slow oscillations in primary sensory areas, with increased low-frequency fluctuations despite reduced external drive (Lemieux et al., 2014; Marschall et al., 2020). In pDOC, “over-activation” in calcarine cortex may therefore reflect disorganized intrinsic activity in a structurally or functionally disconnected region, rather than preserved visual processing. This interpretation is compatible with diffusion MRI and case reports showing damage and delayed reconstruction of visual white-matter pathways, such as the optic radiation and sagittal stratum, during recovery of visual tracking from VS/UWS (Cavaliere et al., 2014; Wijnen et al., 2014).

Several alternative explanations must also be considered. ALFF is sensitive to global arousal level, vascular reactivity, physiological noise and residual head motion (Golestani et al., 2017; Goodale et al., 2021; Horovitz et al., 2008). Fluctuations in vigilance or sedation may alter occipital BOLD power without indicating specific changes in visual information processing, and these factors are difficult to control in severely brain-injured patients. The small sample size further increases the risk that the observed correlation reflects sampling variability. In addition, we did not acquire task-based visual activation data, so it remains unknown whether regions with high resting-state ALFF would show preserved visually evoked responses. For these reasons, the visual cortex findings are exploratory and should be interpreted with caution.

Our results are also in line with, and extend, metabolic and multimodal evidence implicating the visual system in pDOC. FDG-PET studies have reported reduced metabolism in occipital, parietal and temporal cortices and have used these patterns to help distinguish MCS from VS/UWS and to relate occipital uptake to CRS-R scores (Golkowski et al., 2017; He et al., 2024; Liu et al., 2024). DTI studies have demonstrated widespread abnormalities in visual white-matter tracts (Cavaliere et al., 2014), and multimodal or graph-based approaches combining fMRI, DTI and/or PET suggest that features of visual cortices and their large-scale network embedding can aid classification and prognosis (Chen et al., 2021; Golkowski et al., 2017; Qi et al., 2024). However, most larger rs-fMRI and PET studies report overall reductions, rather than focal increases, in occipital activity or metabolism. This discrepancy may reflect methodological differences, heterogeneity in etiology and time post-injury, or, importantly, the very small sample in the present study. Together with our findings, the literature underscores that functional-structural coupling between the occipital visual cortices and large-scale networks such as the DMN and SN plays a pivotal role in determining awareness levels. Taken together, these data support the occipital visual cortices and their connections with higher-order networks as candidate regions for future multimodal work, but larger longitudinal cohorts will be required before specific occipital ALFF patterns can be considered as potential markers of consciousness level in pDOC.

### Aberrant connectivity within core consciousness networks

Resting-state fMRI studies show widespread alterations in networks subserving consciousness in patients with pDOC. Within the DMN, the excitatory-inhibitory balance of the core hub—especially the posterior cingulate cortex (PCC)—is disrupted, with reduced self-inhibition, increased oscillatory power, and impaired effective interactions with prefrontal regions (Chen et al., 2021). Functional coupling between the DMN, SN and executive control network decreases stepwise as behavioral awareness declines (van Son et al., 2019). Additional networks mediating fronto-attentional/executive control, sensorimotor, visual and auditory processing also show significant reductions in connectivity (Demertzi et al., 2015). Conversely, the FPN exhibits pathological hyper-connectivity that is negatively correlated with DMN integrity and becomes more pronounced in patients with lower levels of consciousness, indicating loss of the normal balance between internally and externally oriented systems (Long et al., 2016).

Multimodal investigations reveal decoupling across structural, metabolic and functional domains in pDOC. FDG-PET studies show that restoration of DMN connectivity is paralleled by increases in cortical glucose metabolism, whereas patients who remain unresponsive exhibit reduced metabolism accompanied by aberrant cross-network positive correlations (Di Perri et al., 2016). Ultra-high-field 7 T DTI demonstrates loss of long-range white-matter fibers, with network topology shifting toward higher local clustering and lower global integration; clustering coefficients correlate negatively with consciousness scores (Tan et al., 2019). Metabolically, reduced activity in the central thalamus together with relative hyperactivity of the globus pallidus supports the mesocircuit model and links subcortical under-drive to widespread frontoparietal hypometabolism (Fridman et al., 2014). Combining PET, MRI and EEG in single patients can identify covert but functionally intact networks and improve diagnostic and prognostic accuracy. Integrated assessment of structure, function and metabolism therefore provides a critical window into the mechanisms and monitoring of consciousness recovery.

### White matter integrity vs. functional/metabolic change

Structural white-matter injury is a defining feature of pDOC, yet its relationship with functional and metabolic brain activity is not strictly linear. DTI studies consistently demonstrate widespread reductions in FA throughout major white-matter tracts in pDOC. Patients in an UWS typically show more pronounced FA decreases than those in a MCS, and DTI derived metrics can partially discriminate between these diagnostic categories (Vitello et al., 2022). White matter integrity also possesses prognostic value. In post-anoxic coma, for example, a whole-brain FA below a critical threshold predicted non-recovery with 100% specificity in one cohort study (Kumar et al., 2023; Velly et al., 2018). Likewise, damage to subcortical and callosal fibers has been linked to failure to regain consciousness, whereas preservation of pivotal pathways, especially those within the ascending arousal network, is associated with more favorable outcomes.

However, inconsistencies between white matter damage and functional/metabolic activity are frequently observed, carrying important prognostic implications. Some patients with extensive DTI abnormalities still show islands of functional/metabolic brain activity, suggesting that functional networks can, to a degree, re-route or compensate despite structural lesions. For example, a number of clinically vegetative patients had intact sensory evoked potentials and active cortical metabolism even with presumed axonal injuries, indicating covert preservation of pathways not evident from structural MRI alone (Fernández-Espejo et al., 2012; Zheng et al., 2013). There are also cases of the opposite mismatch. Relatively preserved white matter structure but profoundly depressed cortical metabolism and connectivity (Annen et al., 2016). Because of such discordances, single-modality assessments can be misleading. Multimodal imaging approaches have proven most valuable for prognosis. Studies show that combining metabolic and electrophysiological measures can unveil covert consciousness and improve outcome prediction beyond structural metrics alone. In one recent cohort, the addition of FDG-PET and EEG to clinical exam allowed identification of hidden cognitive processing and significantly improved 6-month outcome predictions for behaviorally unresponsive patients (Hermann et al., 2021). Similarly, diffusion tractography mapping of subcortical connections, when integrated with functional MRI/EEG, can inform whether enough structural substrate exists for recovery (Fernández-Espejo et al., 2010). These findings highlight that structural damage and functional impairment can be decoupled in pDOC.

### Research limitations

Despite the encouraging findings, several methodological constraints temper the strength and generalizability of our conclusions. First, this feasibility study included a small sample (8 pDOC and 8 controls), which limits statistical power, increases uncertainty around effect-size estimates, and precludes robust subgroup analyses. Because lesions were large and heterogeneous, we did not perform lesion-aware normalization or quantitative lesion-result overlap analyses; thus, lesion-related confounding cannot be excluded and attribution of network alterations to consciousness level alone is limited. Accordingly, our findings should be considered exploratory. Second, the cross-sectional design provides only a single temporal snapshot and cannot distinguish state-dependent fluctuations (e.g., arousal variation) from trait-like network alterations. Longitudinal multimodal PET/MR with repeated behavioral assessments is needed to model dynamic trajectories of metabolic and functional reorganization and to relate them to recovery outcomes. Third, the cohort was aetiologically heterogeneous and scanned at variable post-injury intervals. Although such diversity reflects real-world clinical populations, it introduces confounds related to lesion burden, secondary complications, and pharmacological regimens. Rigorous stratification or covariate modeling was precluded by sample size. Thus, our findings should be interpreted as preliminary.

To address these shortcomings, future investigations should: (i) recruit larger, multicenter cohorts with harmonized acquisition protocols; (ii) adopt longitudinal designs to model dynamic network plasticity, including “time-to-consciousness” as an outcome; (iii) incorporate control groups with other severe neurological conditions to refine disorder-specific biomarkers.

### Clinical implications

Simultaneous PET/MR has been proposed as an objective adjunct to behavioral scales in patients with prolonged disorders of consciousness by quantifying cortical metabolism and large-scale network integrity. Large-cohort studies using FDG-PET, sometimes in combination with EEG, have reported that metabolic indices such as the “metabolic index of the best-preserved hemisphere” and disorder-specific uptake patterns can help to distinguish MCS from UWS and to stratify prognosis when combined with CRS-R scores (Guo et al., 2025; Hermann et al., 2021). In parallel, resting-state fMRI measures have also shown prognostic value in disorders of consciousness (Le Cause et al., 2024). Together, these data suggest that advanced imaging markers can complement clinical examination and may reduce diagnostic misclassification in pDOC and related conditions (Farg et al., 2024; Guo et al., 2025; Hermann et al., 2021). However, the present pilot cross-sectional study was not designed to evaluate diagnostic accuracy or prognostic prediction.

Within this exploratory context, regions showing convergent metabolic and functional abnormalities in our cohort, such as the posterior cingulate cortex and anterior insula, may be regarded as candidate targets for future neuromodulation or pharmacological studies, in line with prior work implicating default mode and salience network hubs in prognosis and recovery in disorders of consciousness (Dondi et al., 2025; Song et al., 2018; Zhang et al., 2018). Likewise, the observed dissociation between widespread white-matter damage and partially preserved cortical activity is compatible with diffusion–functional imaging studies showing that severe white-matter disruption can coexist with residual large-scale network integrity and covert information processing (Edlow et al., 2021; Luppi et al., 2021; Snider and Edlow, 2020).

The broader clinical implementation of PET- or PET/MR-based assessment in pDOC is further constrained by practical factors, including scanner availability, costs, radiation exposure and the risks associated with transporting medically fragile patients (Farg et al., 2024). Formal health-economic evaluations and prospective multicenter studies with standardized longitudinal follow-up will be required before the integration of PET/MR into tiered diagnostic or prognostic pathways can be considered. At this stage, the main contribution of the present work is to demonstrate the feasibility of simultaneous PET/MR acquisition in pDOC and to provide preliminary multimodal patterns that may help to design and power future longitudinal and multicentre investigations.

## Conclusion

This pilot feasibility study used simultaneous 18F-FDG PET/MR to provide a preliminary, integrated characterization of functional, metabolic, and structural brain alterations in patients with pDOC. We observed convergent disruptions in key cortical hubs of the default mode, salience and visual networks, with the posterior cingulate cortex and occipital visual cortices emerging as candidate regions whose abnormalities were associated with lower CRS-R scores. At the same time, the dissociation between widespread white-matter injury and partially preserved cortical activity in some patients highlights the limitations of single-modality assessment and illustrates how multimodal imaging can reveal structure-function decoupling in pDOC. Because of the small sample size, etiological heterogeneity, and cross-sectional design, these findings are preliminary and require validation. Future multicenter, longitudinal studies with harmonized protocols are needed to validate these candidate biomarkers and determine their potential to contribute to prognosis and personalized management in pDOC.

## Data Availability

The raw data supporting the conclusions of this article will be made available by the authors, without undue reservation.

## Footnotes

1
http://www.fil.ion.ucl.ac.uk/spm/

2
http://fsl.fmrib.ox.ac.uk/fsl/
