# Supplementary material for: Multimodal PET/MR imaging of prolonged disorders of consciousness: a pilot feasibility study
Source: Front Neurosci. 2026 Feb 20;20:1761097. doi: 10.3389/fnins.2026.1761097 (PMC12963328; doi:10.3389/fnins.2026.1761097)
Supplement: Supplementary file 1 [file Data_Sheet_1.docx]

Table S1. Individual-level clinical confounders and lesion summaries

| Patient | Zolpidem (60 min pre-scan, 10 mg) | Ventricular enlargement / hydrocephalus (Y/N/NA) | Lesion summary (radiology report–based) |
| --- | --- | --- | --- |
| P1 | No | Y | L skull defect; L hemispheric + R frontal hypodensity with SAH/blood; R subdural effusion (~0.8 cm); L ventricular enlargement; aneurysm clip artifact. |
| P2 | No | NA | Large R frontotemporal mass crossing midline; marked edema + corpus callosum involvement; severe ventricular compression (R>L); slight leftward midline shift. |
| P3 | Yes | Y | Postoperative L bone flap; perioperative fluid/pneumocephalus + adjacent hypodensity/edema; ventriculomegaly with periventricular changes; midline centered. |
| P4 | No | NA | L skull defect/meningoencephalocele; L hemispheric hypodensity with SAH/small hematomas; ventricular compression + mild rightward midline shift; small IVH. |
| P5 | No | Y | Post L decompressive craniectomy; extensive L>R encephalomalacia (frontal–basal ganglia–parieto-temporal/insula) with brainstem involvement; mild ventriculomegaly; small subdural effusion. |
| P6 | No | Y | Brainstem + R middle cerebellar peduncle hypodensity; bilateral basal ganglia small infarcts; post burr-hole/drainage; mild ventriculomegaly/periventricular change. |
| P7 | No | NA | R skull defect; extensive R hemispheric hypodensity (F-P-T-O); basal ganglia + brainstem hypodensity; infarct/encephalomalacia considered. |
| P8 | Yes | NA | Bilateral frontal white matter abnormalities; mild brain swelling; EEG diffuse encephalopathy pattern. |


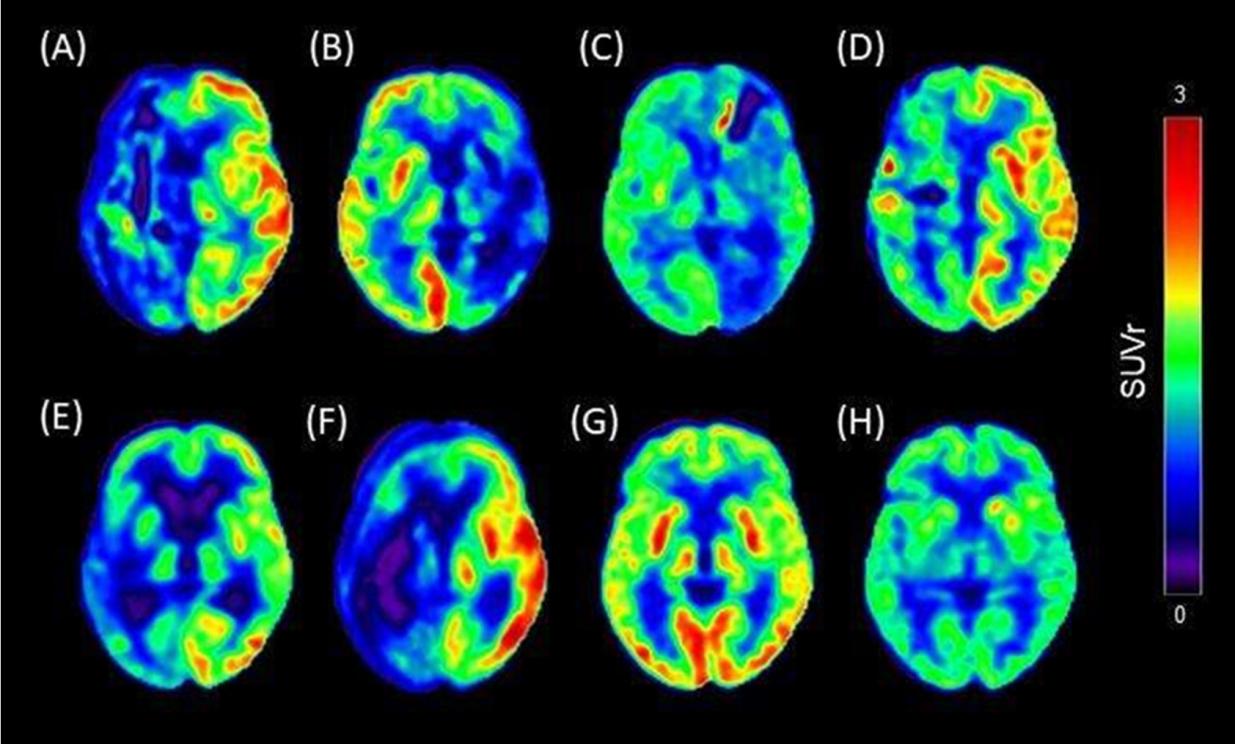


Figure S1. Individual FDG metabolism maps of pDOC patients. Panels (A–H) correspond to patients P1–P8, respectively.
